# Early detection of brain metastases and appropriate local therapy followed by systemic chemotherapy may improve the prognosis of gastric cancer

**DOI:** 10.1038/s41598-023-46933-z

**Published:** 2023-11-27

**Authors:** Yasunobu Ishizuka, Takeshi Omori, Naoki Shinno, Masaaki Yamamoto, Hisashi Hara, Tomoyuki Otsuka, Minako Nishio, Naohiro Nishida, Fumie Fujisawa, Naotoshi Sugimoto, Toshinari Yagi, Masahiro Goto, Hiroki Nishikawa, Toshihiro Kudo

**Affiliations:** 1 Department of Medical Oncology, Osaka International Cancer Institute, 3-1-69, Otemae, Tyuou-ku, Osaka-shi, Osaka 541-8567 Japan; 2 Department of Gastroenterological Surgery, Osaka International Cancer Institute, Osaka, Japan; 3 Cancer Chemotherapy Center, Osaka Medical and Pharmaceutical University Hospital, Osaka, Japan; 4 Second Department of Internal Medicine, Osaka Medical and Pharmaceutical University Hospital, Osaka, Japan

**Keywords:** Cancer, Oncology

## Abstract

Brain metastases develop in 0.5–0.7% of patients with gastric/gastroesophageal junction (G/GEJ) cancer. Although rare, brain metastasis is often identified when the patient is already symptomatic; hence prognosis is poor. Given the therapeutic developments for G/GEJ cancer, overall survival is prolonged, thereby the incidence of brain metastases is predicted to increase. We retrospectively surveyed the rate of brain metastasis among 1257 patients diagnosed with G/GEJ cancer who received chemotherapy between January 2011 and April 2021. We investigated the time of onset of brain metastasis, treatments administered, and impact of the metastasis on the overall treatment course and prognosis. Of the 741 patients included in the analysis, brain metastasis was confirmed in 16 (2.2%). The median survival time (MST) from G/GEJ cancer diagnosis was 14.9 months in patients with brain metastasis detected during the treatment period, and the MST from the diagnosis of brain metastasis was 2.8 months. Patients who received chemotherapy exhibited prolonged survival compared with those who did not (12.4 months vs 1.0 months, *p* < 0.001). Our findings suggest that the early detection of brain metastases and local therapy for poor responders to chemotherapy enable the continuation of chemotherapy and prolong survival.

## Introduction

Gastric cancer is the fifth most common malignancy and the fourth leading cause of cancer-related deaths worldwide; furthermore, the incidence and mortality rates of gastric cancer are highest in Eastern Asia^1^. Gastric/gastroesophageal junction (G/GEJ) cancer frequently metastasizes to various organs and is associated with a poor prognosis. The most common organs for metastasis to occur in association with G/GEJ cancer are the liver (48%), peritoneum (32%), lungs (15%), and bones (12%)^2^.

The brain is a relatively rare organ to which G/GEJ cancer metastasizes, with a prevalence of approximately 0.5–0.7% based on reports from 1999 and 2000^3,4^. Brain metastases in G/GEJ cancer tend to be identified owing to associated symptoms in the latter half of the course of treatment^5^. However, the reason for the low incidence of brain metastasis in patients with unresectable advanced or recurrent G/GEJ cancer may be that the overall survival (OS) is short, even following treatment with systemic chemotherapy, such that patients die before brain metastases occur.

Combination therapy of fluorinated pyrimidines and platinum-based drugs is currently used as the first-line systemic chemotherapy treatment for unresectable advanced or recurrent G/GEJ cancer. Until the year 2000, 5-fluorouracil/cisplatin was used for treatment, with a median OS of 9.3 months^6^. However, with advances in drug development, the concomitant use of angiogenesis inhibitors as second-line treatments^7^, as well as immune checkpoint inhibitors (ICI) and trifluridine/tipiracil (FTD/TPI) as third-line treatments, has gradually prolonged the OS of patients with G/GEJ cancer^8,9^. Indeed, the phenomenon of G/GEJ cancer having an OS of less than 1 year is becoming less common globally, as the combined use of cytotoxic anticancer drugs and ICI as first-line treatments has resulted in prolonged survival^10^. Furthermore, the advent of trastuzumab and the human epidermal growth factor receptor 2 (HER2)-targeting antibody–drug conjugate trastuzumab deruxtecan for HER2-positive G/GEJ cancer, has improved the prognosis of HER2-positive G/GEJ cancer^11,12^. As for other types of cancer, biomarker-oriented therapy, underscored by the proven efficacy of ICI for microsatellite instability-high (MSI-H) G/GEJ cancer, has been developed^13,14^. The incidence of brain metastasis, in turn, may be increasing owing to prolonged survival associated with novel drug development. Although a report from Surveillance, Epidemiology, and End Results indicates that the rate of brain metastasis from G/GEJ cancer is approximately 0.6%, details on the time from the diagnosis of gastric cancer to the diagnosis of brain metastasis, prognosis after the diagnosis of brain metastasis, and treatment administered for brain metastasis are lacking^15^.

Brain metastases are often identified in the form of neurological symptoms such as limb paralysis and psychiatric symptoms such as dementia^4^. These symptoms cause a decrease in performance status, rendering the continuation of chemotherapy difficult. In many cases, the symptoms of brain metastasis are the vital determinants of prognosis, and patients with brain metastases tend to have a poor prognosis. In modern times, with advances in the pharmacological treatment of G/GEJ cancer, the ability to control brain metastases is still considered a matter of clinical importance. Much is still unknown about the incidence and characteristics of brain metastases and their treatment course in patients undergoing systemic chemotherapy. The purpose of this study was to clarify the factors that support the continuation of chemotherapy for G/GEJ cancer with brain metastasis as long as possible. To achieve this, we retrospectively analyzed G/GEJ cancer cases in our hospital, examining the timing of brain metastasis occurrence, specific treatments administered for brain metastasis, and impact of brain metastasis on participation in subsequent systemic chemotherapy. The clinical characterization of the timing and course of brain metastases may help prevent the unexpected discontinuation or interruption of systemic chemotherapy due to brain metastases, which in turn, may lead to longer OS.

## Results

### Patient characteristics

**Figure 1:**
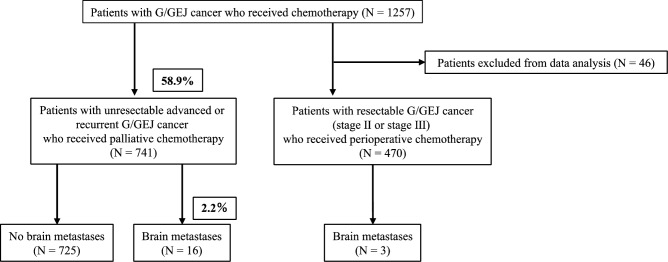
Flowchart of the selection process of brain metastasis cases from cases receiving chemotherapy for gastric/gastroesophageal junction cancer between 2011 and 2021. G/GEJ: gastric/gastroesophageal junction.

Of the 741 patients who received chemotherapy for unresectable advanced or recurrent G/GEJ cancer, 16 (2.2%) were confirmed to have brain metastases (Fig. 1). In addition, three cases of recurrent brain metastases were observed among the 470 patients who received only chemotherapy in the perioperative period. No differences were observed in patient characteristics or incidence of brain metastasis among the time period groups (Table 1). Three cases of recurrent postoperative brain metastases were included, one in each time period.

**Table 1:** Patient characteristics for each time period.

	Total (N = 741) (%)	A (N = 217) 2011–2014	B (N = 293) 2015–2018	C (N = 231) 2019–2021	P
Median age, years (range)	68 (22–90)	68 (22–84)	69 (26–87)	68 (28–90)	–
Sex (male/female)	488/253 (66/34)	146/71 (67/33)	192/101 (66/34)	150/81 (65/35)	0.86
Metastatic/recurrence	549/192 (74/26)	159/58 (73/27)	210/83 (72/28)	180/51 (78/22)	0.25
HER2 (+/−/unknown)	121/553/70 (16/75/9)	21/134/62 (10/61/29)	55/236/2 (19/80/1)	45/183/3 (20/79/1)	–
Brain metastases	16 (2.2)	3 (1.4)	7 (2.4)	6 (2.6)	0.63

HER2: human epidermal growth factor receptor 2.

Liver and lung metastases were observed in six (37.5%) of the 16 patients with synchronous or metachronous distant metastases, respectively. Bone metastases were only observed in two patients (12.5%). The median number of brain metastases was two, and the median maximum diameter of the metastases was 21 mm. Metastasis to the cranial side of the diaphragm was noted in 13 patients (81.2%; Table 2). Since nivolumab is available as a third line or later treatment, the MSI statuses were only in one of the studied cases. Furthermore, since the study focused on cases before programmed cell death ligand 1 (PD-L1) status measurement initiation, none of the PD-L1 status was measured.

**Table 2:** Characteristics of patients with brain metastases.

	Number of patients (N = 16)	Percent (%)
Median age (range), years	71 (41–78)	–
Sex		
Male	11	69
Female	5	31
Histological type^a^		
Intestinal	10	–
Diffuse	13	–
HER2 status		
Positive	5	31
Negative	11	69
Macroscopic type		
Type 1	0	0
Type 2	4	25
Type 3	6	38
Type 4	2	12
Unknown	4	25
Primary tumor location		
Gastroesophageal	3	19
Cardia	2	12
Corpus	9	57
Pyloric antrum	2	12
Site of metastases		
Liver	6	38
Lung	6	38
Lymph node	12	75
Peritoneal dissemination	4	25
Bone	2	13
Metastases above the diaphragm		
+	13	81
−	3	19
MSI high		
Yes	0	0
No	1	6
Unknown	15	94
Neurological symptoms		
+	13	81
−	3	19
Treatment of brain metastases^b^		
Surgery	4	25
SRT	7	44
WBRT	2	13
BSC	3	19
Treatment line when brain metastases were diagnosed		
0	3	19
1	5	31
2	5	31
3	3	19

Data are presented as no. (%) unless otherwise indicated.

HER2: human epidermal growth factor receptor 2, SRT: stereotactic radiotherapy, WBRT: whole-brain radiation therapy, BSC: best supportive care.

^a^Pathology findings include duplicates. ^b^Cases with SRT after surgery are included in the surgery group.

Thirteen patients (81.3%) were diagnosed with brain metastasis after the appearance of symptoms, and three (18.7%) were asymptomatic. Despite some overlap, the following symptoms were observed: staggering (n = 3), vertigo (n = 2), cognitive decline/altered consciousness (n = 4), limb weakness (n = 3), quadriplegia (n = 2), nausea/vomiting (n = 3), dysarthria (n = 1), and constriction of the visual field (n = 1). Two of the three asymptomatic patients were observed to have brain metastasis based on head computed tomography (CT) scans obtained as part of screening, whereas the remaining patient was observed to have brain metastasis based on magnetic resonance imaging (MRI) scans obtained after a temporary loss of consciousness due to the vagal reflex, and not brain metastasis. Two (12.5%) of the patients were diagnosed with brain metastasis upon initial examination, and 14 (87.5%) were diagnosed with brain metastasis over the course of systemic chemotherapy.

### HER2-positive cases

The incidence of brain metastases in HER2-positive patients tended to be higher than that in HER2-negative patients, although this was not significant (HER2-positive: 4.2%; HER2-negative: 2.4%, *p* = 0.26).

**Figure 2:**
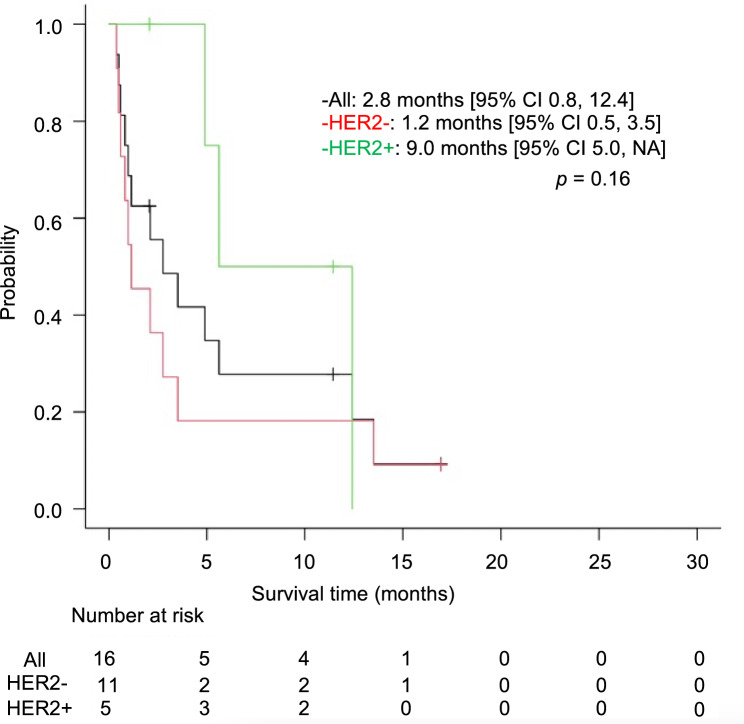
Kaplan–Meier analysis of overall survival for patients with brain metastasis after brain metastasis diagnosis. Survival after diagnosis of brain metastases according to HER2 status. HER2: human epidermal growth factor receptor 2, NA: not applicable.

Among the patients with brain metastasis, five of 16 patients (31.3%) were HER2-positive. All of these patients received trastuzumab as first-line treatment; two patients received trastuzumab deruxtecan as third-line or later treatment. The median time from G/GEJ cancer diagnosis to brain metastasis, according to HER2 status, was 10.9 and 13.1 months for HER2-positive and HER2-negative patients, respectively; there was no significant difference between the groups (*p* = 0.41). After brain metastasis diagnosis, the median survival time (MST) was 9.0 and 1.2 months for HER2-positive and HER2-negative patients, respectively. Although the MST was longer in HER2-positive cases, there was no significant difference, and HER2-positive brain metastases did not confer a favorable prognosis (*p* = 0.16, Fig. 2).

### Systemic chemotherapy

With the exception of one elderly patient who received fluorinated pyrimidine monotherapy, all patients received treatment with fluorinated pyrimidine plus platinum-based anticancer drugs as first-line treatment. Taxane-based treatments were administered as second-line treatment. Nivolumab, FTD/TPI, and irinotecan were used as third-line and subsequent therapies. There were five patients to which ICIs were administered, and nivolumab was used as a third-line therapy or later. Table 3 lists the treatment regimen for each case.

**Table 3:** Treatment and outcome of patients with brain metastases.

Case	Age	Sex	HER2 status	Treatment regimen				Treatment line brain metastases were diagnosed	Treatment of brain metastases	Survival after diagnosis of brain metastases (months)
				1st line	2nd line	3rd line	4th line			
1	75	M	+	XP + Tmab	PTX			0	SRT	12.4
2	63	M	−	SP	PTX			2	BSC	0.8
3	74	M	−	XP	PTX + study drug			2	BSC	0.6
4	70	M	+	XP + Tmab	PTX + RAM	NIVO	CPT-11	3	SRT	4.9
5	41	F	−	XELOX	Nab-PTX + RAM	NIVO	CPT-11	3	Surgery	16.9
6	71	F	−	XELOX	Nab-PTX			2	WBRT	1.2
7	75	M	−	XELOX	Nab-PTX + RAM	FTD/TPI	NIVO	1	Surgery	13.5
8	68	M	−	SOX				1	BSC	1.0
9	76	M	−	SOX				1	Surgery	0.4
10	76	M	−	XELOX	PTX + RAM	NIVO		3	SRT	2.8
11	64	M	+	XELOX + Tmab				1	SRT	11.4
12	56	F	−	SOX				0	Surgery	2.1
13	71	F	+	XELOX + Tmab	Nab-PTX + RAM	NIVO	T-Dxd	2	SRT	5.6
14	62	M	−	XELOX	PTX + RAM			2	WBRT	0.5
15	78	M	−	S1				0	SRT	3.5
16	73	M	+	SOX + Tmab	PTX	T-Dxd		1	SRT	2.1

HER2: human epidermal growth factor receptor 2, XP: capecitabine + cisplatin, Tmab: trastuzumab, XELOX: capecitabine + oxaliplatin , SOX: S1 + oxaliplatin, PTX: paclitaxel, RAM: ramucirumab, NIVO: nivolumab, CPT-11: irinotecan, T-Dxd: trastuzumab deruxtecan, SRT: stereotactic radiotherapy, WBRT: whole-brain radiation therapy, BSC: best supportive care.

### Prognosis of patients with brain metastases

**Figure 3:**
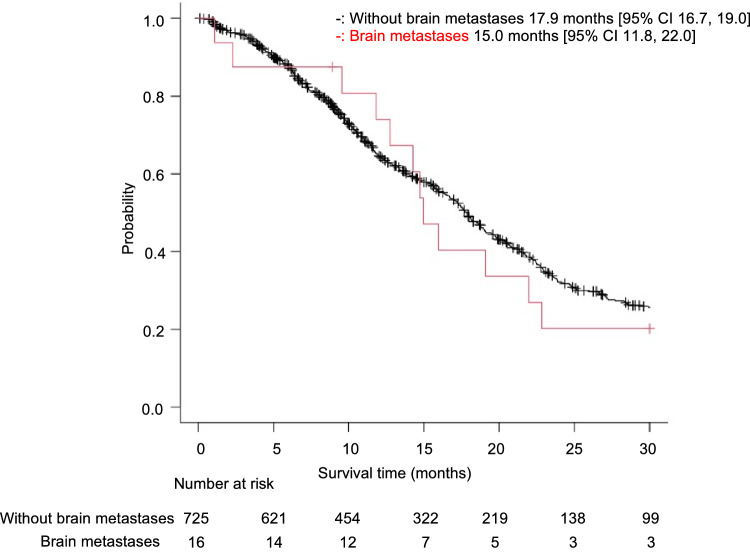
Kaplan–Meier curve depicting the overall survival of gastric cancer cases from the time of gastric cancer diagnosis. CI: confidence interval.

**Figure 4:**
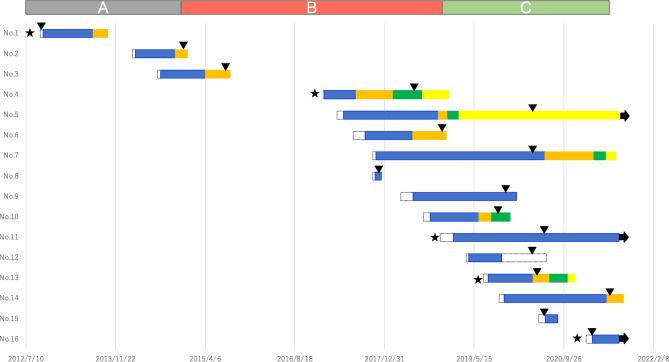
Treatment course and time of emergence of brain metastases. Black filled down pointing triangle: diagnosis of brain metastases, arrow: alive, star: HER2+, blue filled square: 1st line, orange filled square: 2nd line, green filled square: 3rd line, yellow filled square: 4th line and beyond. Patients with recurrence of brain metastases who were cured by surgery and patients with recurrence of brain metastases after perioperative chemotherapy were excluded.

**Figure 5:**
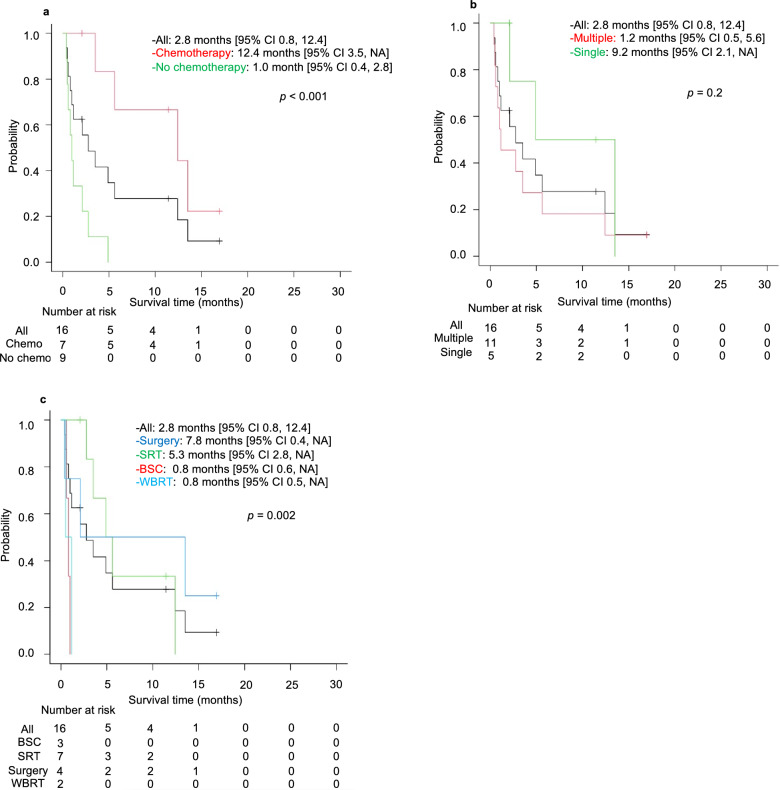
Kaplan–Meier analysis of overall survival for patients with brain metastasis after brain metastasis diagnosis. (**a**) Survival after diagnosis of brain metastases for patients undergoing or not undergoing chemotherapy. (**b**) Survival after diagnosis of brain metastases according to number of brain metastases. (**c**) Survival after diagnosis of brain metastases according to treatment for brain metastases. SRT: stereotactic radiotherapy, WBRT: whole-brain radiation therapy, BSC: best supportive care, CI: confidence interval, NA: not applicable.

The median OS for patients with and without brain metastasis was 15.0 and 17.9 months, respectively; there was no significant difference between groups (*p* = 0.63, Fig. 3). The median duration from G/GEJ cancer diagnosis to brain cancer diagnosis was 12.9 months. After brain metastasis diagnosis, the MST was 2.8 months, indicating a poor prognosis. Furthermore, we identified that brain metastases developed not only late in the course of the whole treatment but also in several patients with long progression-free survival following first-line therapy who died early after the emergence of brain metastases (Fig. 4). Of the patients, seven could and nine could not receive chemotherapy after achieving local control of brain metastasis by therapeutic intervention. Patients who could receive chemotherapy exhibited significant prolongation of survival compared with patients who could not (12.4 months vs. 1.0 month, *p* < 0.001, Fig. 5a).

When classified by the number of brain metastases, five patients had a single lesion and 11 had multiple lesions (patients with meningeal dissemination alone were classified as having multiple lesions). The MST from brain metastasis diagnosis exhibited a trend to be longer in the single-lesion group than in the multiple-lesion group, but this was not significant (9.2 vs. 1.2 months, respectively; *p* = 0.2, Fig. 5b).

With regard to categorization by treatments received, four patients underwent surgery, seven underwent stereotactic radiotherapy (SRT), two underwent whole brain radiotherapy (WBRT), and three received best supportive care (BSC) without therapeutic intervention for brain metastases. The MST from brain metastasis diagnosis was 7.8, 5.3, 0.8, and 0.8 months for patients in the surgery, SRT, WBRT, and BSC groups, respectively (*p* = 0.002, Fig. 5c). There was no significant difference in survival time between patients receiving WBRT and those receiving BSC (*p* = 0.6). Two patients in the surgery group and five in the SRT group could continue chemotherapy after being diagnosed with brain metastases. In contrast, none of the patients in the WBRT and BSC groups could receive chemotherapy, and all patients died owing to brain metastases.

## Discussion

To the best of our knowledge, this is the first study to investigate the incidence of brain metastases in a population of G/GEJ cancer cases with distant metastases, or recurrent cases requiring palliative chemotherapy. We observed that the incidence of brain metastasis was 2.2% in our institution. Although our study examined brain metastases in patients who were candidates for systemic chemotherapy, previous reports examined brain metastases in all cases diagnosed with G/GEJ cancer, including those that could be curatively resected^3,4,16,17^. Therefore, when comparing study findings, it is necessary to pay attention to this difference.

In reports published between 2018 and 2021, the rate of brain metastases has been reported as 2.3–6.5%^16–18^, highlighting the rising prevalence of brain metastases, given that older reports from around the year 2000 reported incidence rates of 0.4–0.7%^4,6^. Assuming that the probability of the emergence of brain metastasis per unit time is constant, the longer the survival period, the greater the chance a patient will develop brain metastasis. In other words, the higher incidence of brain metastases in recent reports than in older reports may be due to the prolongation of OS from advances in anti-cancer drugs. Another possible reason is that, because the brain has a blood–brain barrier, it is difficult for anti-cancer drugs to reach brain metastases, making them resistant to pharmacotherapy. It is also possible that the frequency of brain metastasis has increased. In contrast to our hypothesis, we did not observe an increase in the prevalence of brain metastases over time when dividing the investigation period into phases of approximately 4 years each, from 2011 onward. It is possible that we did not observe a difference because we were only able to accumulate cases over a 10-year period in this study. In consideration of the fact that the OS of G/GEJ cancer cases was approximately 7–9 months during the period from 1990 to approximately 2000^6,19^, and that more brain metastasis cases are diagnosed after the 7- to 9-month timepoint, the finding of 12/16 cases in this study developing brain metastases after 9 months, at least partially supports our hypothesis.

We observed several patients (including Cases 2, 3, 6, 9, and 14) who developed brain metastases after relatively long disease control with continued first- or second-line chemotherapy of nearly 1 year and died early after failure to control brain metastasis. It is regrettable that patients are dying from brain metastasis prior to being able to exhaust all available anti-cancer drugs, especially given the increase in treatment options. Therefore, if a patient is receiving prolonged first- or second-line treatment, it may be possible to diagnose brain metastasis in a still-controllable state if the patient undergoes brain metastasis screening at a certain timepoint (e.g., at approximately 12 months of treatment).

Human epidermal growth factor receptor 2-positivity in G/GEJ cancer has been reported in approximately 17.7% of all cases^20^. The HER2-positivity rate among brain metastasis cases at our hospital tended to be higher at 28%. Although it is difficult to say that the rate of complication of brain metastasis in HER2-positive G/GEJ cancer is high based on this alone, this result indicates that the rate of HER2-positive G/GEJ cancer is numerically high. Breast cancer is associated with high incidence rates of brain metastasis among HER2-positive patients^21^, and some reports have indicated similar results in HER2-positive patients with G/GEJ cancer^22,23^. It has been postulated that some biological characteristics are the reason for the high incidence of brain metastasis in HER2-positive tumors, but the detailed underlying mechanism is unknown. However, one of the most plausible reasons is that, although trastuzumab has a high anti-tumor effect against HER2-positive tumor cells, it is an antibody drug with a large molecular weight that makes it difficult to pass through the blood–brain barrier to control metastasis. As a result, it is thought that this may increase the complication rate of brain metastases in HER2-positive cancers^24^. Moreover, one report suggests that HER2-positive cases are associated with a shorter period of time from G/GEJ cancer diagnosis to the emergence of brain metastases than HER2-negative cases^23^, and there is a similar report in breast cancer^25^. However, the median time between G/GEJ cancer and brain metastasis diagnoses in our study was 10.9 months, which was not significantly different from the median time of 13.1 months for HER2-negative cases. As the study at our hospital comprised a small number of cases (n = 5), it is necessary to accumulate additional cases in the future, considering the possibility that the incidence of brain metastasis is high in HER2-positive G/GEJ cancer and screening examination for brain metastasis may be necessary from an early stage.

Brain metastasis from G/GEJ cancer is often diagnosed in the symptomatic stage, and the survival time from G/GEJ cancer diagnosis is reported to be 2–4 months, indicating a poor prognosis^3,4,6,17^. In this study, the MST from brain metastasis diagnosis was 2.8 months, which is similar to those in these previous reports. However, when we looked at the course of each case, it became clear that the prognosis varied depending on the treatment administered for brain metastases. In other words, in cases for which brain metastasis could be locally controlled by SRT or surgery, chemotherapy could be continued thereafter, and the progression of metastatic lesions in organs other than the brain tended to be the direct cause of death. This suggests that the appropriate management of brain metastasis permits the continuation of systemic chemotherapy and prolongs survival. The MST from brain metastasis diagnosis was 7.8, 5.3, 0.8, and 0.8 months in the surgery, SRT, WBRT, and BSC groups, respectively. These findings indicate that treatment with WBRT alone does not improve survival time compared with treatment with BSC. Survival time according to the type of brain metastasis treatment was the longest in the surgery group, but the proportion of patients who could receive chemotherapy after local treatment was higher in the SRT group. A study reported that approximately 20% of patients with metastatic brain tumors who undergo SRT, die as a direct result of brain metastases^26^. This suggests that, even in patients with distant metastases to other organs, local control of brain metastases by SRT and surgery followed by appropriate systemic chemotherapy are critical for improving prognosis.

There are some limitations in our study to consider. First, this was a retrospective study. Second, it was conducted at a single institution, and we analyzed only 16 cases. Third, only symptomatic cases of brain metastasis had been evaluated, and we may have missed asymptomatic cases. To overcome these limitations, conducting a prospective, multicenter observational study with periodic screening tests to detect brain metastases is ideal.

Despite believing that the prevalence of brain metastases in patients with G/GEJ might be increasing, this trend was not observed during the time period of this study. Over the course of systemic chemotherapy, early detection and local therapy of brain metastases that respond poorly to chemotherapy may enable the continuation of chemotherapy and prolong survival. Therefore, it is essential to perform multimodal treatments combining surgery, radiotherapy, and chemotherapy, even in patients with brain metastasis. In the case of gastric cancer, which is associated with a gradual increase in survival, as the number of active drugs increases, the appearance of brain metastases does not necessarily mark the end of treatment efforts, but may be just one clinical difficulty that should be overcome.

## Methods

### Patient population

We searched the electronic medical records at the Osaka International Cancer Institute for patients listed as having G/GEJ cancer and receiving chemotherapy regimens for G/GEJ cancer, between January 2011 and April 2021. We identified 1257 cases: among them excluded (n = 46) were patients who underwent chemotherapy for neuroendocrine tumors/cancers, squamous cell carcinoma, carcinoma of unknown primary, and duodenal/small bowel cancer, and those who were diagnosed with G/GEJ cancer and planned to undergo chemotherapy, but whose management plan was changed to BSC.

Only adenocarcinoma cases were examined. After excluding patients who had received adjuvant chemotherapy for Stage II or III conditions, the final sample included 741 patients who had received chemotherapy for unresectable advanced or recurrent G/GEJ cancer. This included patients who had liver or distant lymph node metastases that were resectable, as well as patients who had undergone adjuvant chemotherapy.

For this study informed consent has been waived by Osaka International Cancer Institute Institutional review board (IRB)/ethics committee due to the anonymity and retrospective nature of the study. An information disclosure document that was approved by the Ethics Review Committee of our hospital was published on the hospital’s website. The information disclosure document guarantees research participants and others the opportunity to refuse the implementation or continuation of research. In addition, this study was approved by the Ethical Review Committee of the Osaka International Cancer Institute (No. 22032-2) and conducted with the research permission of the President. All methods were performed in accordance with relevant guidelines and regulations.

### Definition of time periods

With reference to approval timing of several clinically significant drugs in Japan, we divided 741 patients into three distinct period groups. Group A comprised cases associated with the time period since the year trastuzumab was approved (January 2011–December 2014); Group B, the period since the year ramucirumab was approved (January 2015–December 2018); and Group C, the time period since salvage-line treatments such as nivolumab and FTD/TPI became established (January 2019–April 2021).

### Treatment and follow-up strategy

Disease staging was based on findings from upper gastrointestinal endoscopy, CT, and positron emission tomography (PET)-CT scans, using the TNM Classification 8th Edition. Head CT and MRI were performed only for patients with symptoms suggestive of brain metastasis, such as headaches, persistent nausea, dysarthria, and hemiplegia. Therefore, not all cases underwent head screening tests. Tissue collection by biopsy or surgery was not required if the CT or MRI scans determined that the G/GEJ cancer had metastasized to the brain. When possible, lumbar puncture was performed in patients suspected of meningeal dissemination.

### Statistical analysis

Patient characteristics are presented in terms of frequencies (%) or median values (ranges) for continuous variables. Overall survival was defined as time from the first administration of chemotherapy to death by any cause. Monitoring was ceased if the patient was alive on the date of final confirmation. We conducted Chi-squared tests to evaluate the significance of two different groups. A *p*-value < 0.05 indicated significance. Overall survival is illustrated using Kaplan–Meier curves. The significance of the survival curves was assessed using the log-rank test. All statistical analyses were performed with EZR (Saitama Medical Center, Jichi Medical University, Saitama, Japan), which is a graphical user interface for R (The R Foundation for Statistical Computing, Vienna, Austria).

## Data Availability

The datasets used and analyzed during the current study are available from the corresponding author on reasonable request.
